# Transactions of the Pathological Society of Philadelphia

**Published:** 1839-12-07

**Authors:** 


MEDICAL EXAMINER.

DEVOTED TO MEDICINE, SURGERY, AND THE COLLATERAL SCIENCES.

No. 49.] PHILADELPHIA, SATURDAY, DECEMBER 7, 1839. [Vol. II.

### TRANSACTIONS OF THE PATHOLOGICAL SOCIETY OF PHILADELPHIA.

*December 1st*, 1839.

The President, Dr. Gerhard, in the Chair.

Dr. Stillé presented a specimen of *Diseased Heart and Liver*, and read the following account of the case:—

*A case of Ascites, with Disease of the Heart and Liver.*

Margaret E., æt. 35, a native of Germany, and a domestic, entered the Pennsylvania Hospital on the 7th of September last. On the 19th of the same month, the following note was made of her history and condition, by Dr. Stewardson, then attending physician of the hospital. She was married at the age of fifteen; had had six children; all her labours were difficult, the first only instrumental. Two days after the birth of her third child she had a very copious flooding,—and about two years afterwards, at a time when she was not pregnant, experienced a hæmorrhage from the womb in consequence of falling down stairs, by which her right side was injured, but no bones fractured. The flow of blood lasted for three days; at the end of three weeks she was able to sit up, and in three months had entirely regained her health. She had two labours afterwards, both without notable accident, and continued perfectly well until about the month of March last, when, a week before lying in, she was immersed in cold water during a freshet of the Schuylkill, and had a chill, with dyspnœa, but no pain. Her labour was tedious, but terminated without accident: about three weeks afterwards she had an abscess of the mamma, and in the following week a violent cough, with sharp pain at the xiphoid cartilage, extending towards the right side, and over the region of the liver. In about a fortnight she began to observe a tumefaction of the epigastrium. Her physician told her it arose from the liver. For about a month the swelling was confined to the region mentioned, and was then accompanied by a general enlargement of the abdomen, and œdema of the lower extremities.

At the date of this note her intelligence was perfect; her tongue moist, slightly coated with a whitish fur; her pulse quick and feeble. The breathing was short and difficult; there was a slight dry cough, and some fluttering about the heart, noticed about seven months before. Percussion anteriorly was resonant to within one and a half inches of the right false ribs, while on the left side it was so only to within two inches and a half of the nipple. The sounds of the heart were loudest at a point midway between the sternum and the left nipple, and half an inch below the latter. A *bruit de soufflet* existed in the first sound. The abdomen was excessively distended; its skin tense; fluctuation very distinct; soreness on pressure very great for three inches around the umbilicus; pressing the fingers suddenly downwards below the right false ribs, they encountered a firm, resisting body, extending three inches to the left of the xiphoid cartilage, and to the same distance below it, and having a regular and defined edge.

On the 29th September the patient was seized with a severe convulsion, accompanied by twitchings, delirium, and a slow, sighing respiration. Some scarified cups were applied to the back of the neck, and sinapisms to the lower extremities. The relief afforded was immediate. The general treatment of this case consisted in purgation by cream of tartar and jalap, and the use of an infusion of juniper berries. The distention of the abdomen, however, increased. On the 19th October the patient was tapped, and about four gallons of a light yellowish fluid drawn off. Her condition was for a short time rendered more comfortable,—but the dropsy returned, unaccompanied by œdema of the lower extremities, and symptoms of suffocation began to show themselves.

From the beginning of November the patient was under my care. About the 10th of the month she was unable to breathe except in a half-sitting posture; she complained of a feeling of great oppression in the region of the heart, and of tenderness over the whole abdomen; her pulse was small, quick, and corded, and her hands and feet cool. At this time a strong blowing sound accompanied the impulse of the heart, taking the place of the first sound; the second sound was clear and flapping. In a day or two the lips and cheeks acquired a violet colour; the eyes stood staring out; the pulse became slow and jerking, and the inspiration gasping. These symptoms varied in intensity from time to time; and when at their worst, the first sound of the heart was dull, but still marked by a *soufflet*, and the second sound either faint or inaudible. These symptoms daily assumed a new degree of severity, and on the 18th November the patient expired in a state of suffocation.

*Autopsy fourteen hours after death.*—Body of a pale yellow colour; emaciation and rigidity moderate; no œdema of feet; trunk two feet and nine inches in circumference over abdomen, which contained several gallons of yellowish serum; stomach and large intestine much distended by gas; small intestine very much contracted; the peritoneum throughout of a rosy hue, and the veins of the stomach and mesentery very prominent. There was no recent false membrane, but several old adhesions of the colon to the under surface of the liver; the spleen was about four inches long, firm, and its capsule wrinkled; kidnies both of ordinary size and consistence, but of a darker colour than usual; bladder small and contracted; uterus natural. The liver was but little, if at all, larger than natural, but very heavy, and firm. An incision into it was followed by the escape of a considerable quantity of dark blood, particularly when any of the veins were divided. The surface of it was mottled by an infinite number of red and brownish points, visible through the peritoneum. By firm pressure, the finger could be forced into its substance, which was, however, more coherent than natural. A section looked granular in certain lights, but scarcely offered any irregularity to the touch. It presented two distinct colours; the one of venous blood existing in points either isolated or grouped together, or else in lines so disposed as to give the portions thus coloured an arborized appearance. This colour disappeared entirely on maceration. The other was of a pale brown tint, persisting unchanged after repeated maceration and washing in water; this was the colour of the mass of the organ, which was evidently made up of rounded grains, whose sections frequently displayed a central point having three lines radiating from it to the periphery of the acinus. The gall bladder was very much distended with bile of a tarry consistence and colour, in mass, but tinging the skin of a bright orange hue. The pleural cavities contained each about one quart of fluid. The lungs were not attached to the ribs at any point; but on the surface of the left there were several patches of yellowish fibrine, about half a line thick, and slightly adherent. The tissue of the lungs was filled with air, and firmer than natural. The pericardium contained about an ounce of serum; its surface was smooth, and without injection.

The heart was distended by a large quantity of very black, coagulated blood, as were also the ascending and descending cavæ, and the hepatic veins. All the cavities of the heart contained masses of yellow, firm, adherent fibrine, which were largest in the ventricles, and, from them, prolonged into the arterial and auricular orifices. The texture of the heart was firm; remarkably so in the walls of the left ventricle. The extreme length of the heart was five and a half inches; its greatest circumference twelve inches. The right ventricle measured four inches from its point to the origin of the valves of the pulmonary artery; its walls measured from one to three lines in thickness, exclusive of the columnæ carneæ, which were remarkably numerous, thick, and firm. This ventricle was much dilated, as well as the corresponding auricle, which might easily have held a large hen's egg. The tricuspid valve was remarkably well developed; and the *chordæ tendineæ* were not only attached to its free edge, but ran in divergent lines across its expansion, to connect themselves with the muscular margin of the *osteum venosum.*

The walls of the left ventricle were from three to nine lines in thickness, inclusive of the *columnæ carneæ*, which were few in number. Its cavity was three inches and a quarter long, and the side of it towards the right ventricle two inches and a quarter wide. The circumference of the auriculo-ventricular orifice was forty-two lines. Of the two folds of the mitral valve only one could be fairly said to exist, of a trapezoid form, and measuring nine lines from its free edge to its base, and one-third of its length from the latter fourteen lines in width, representing a surface of one hundred and twenty-six square lines; while the plane of the *osteum venosum* being equal to nearly the square of one-third of its circumference, may be valued at two hundred and six square lines. The lesser fold of the mitral valve measured about twelve lines long, by three broad, and its surface thirty-six square lines. So that the aggregate surface of both folds of this valve may be reckoned at one hundred and twenty-six square lines, while the space they should have closed contained two hundred and six square lines. Consequently, the deficiency of valvular apparatus may be valued at forty-four square lines.

The left auricle was dilated, and its appendix, shaped like the finger of a glove, was one inch long. The aorta and pulmonary artery were of their natural size, and their valves perfectly complete and flexible.

The lining membrane of the left cavities of the heart was of a straw-colour, and thicker than natural, but offered no irregularities of any kind. On the mitral valve the same colour and increased thickness were observable towards its free edge, which was of a bright red hue in the breadth of one line all along it margin.

*Remarks.*—The very insufficient details of the symptoms presented during life, in this case, render an intelligible recapitulation difficult. The dates of the origin of the several symptoms which clearly belonged to the lesions, found after death, are almost entirely wanting. The affection of the heart was of a nature which must, in all probability, have given rise to symptoms of a decided character long before the month of March last, from which our history dates the commencement of the disease which ended in death. The affection of the liver is referred to a period, a month or more, after the patient's confinement, and her exposure to cold and wet; so that we are left in the dark relative to the immediate cause of the liver's disease.

It is, indeed, possible that the effects of the cold water, which she describes as being a chill and dyspnœa, without pain, may have been symptomatic of an inflammation of the right lung, subsequently propagated to the liver; but how are we to account for the absence of all symptoms indicative of such inflammation during a period of five weeks, unless by admitting the rather untenable supposition thatthe pains of labour, and of a mammary abscess, were sufficient to mask the symptoms of a pneumonia or a hepatitis? The coincidence of disease of the liver and of the alteration described in this paper, with inflammation and organic disease of the heart, has now been shown in three cases presented to the Society; in all of these cases there was dropsy arising, apparently soon after the first indications of change in the liver, but in all of them there was also disease of the heart, of a date certainly not posterior to that of the liver, and, (if one may estimate the duration of disease by the extent of its lesion,) in the present case probably anterior to the hepatic affection. It remains, then, for new observations to show how far the alterations of the liver and of the heart depended on one another, and whether the dropsical effusion should be referred to either separately, or to both combined. The nature of the lesion of the liver does not appear to be very doubtful; the rounded bodies with a central point could only be the *acini* of this organ, and the surrounding substance, engorged with blood, its cellular parenchyma; so that we may consider the disease a hypertrophy of the glandular structure of the liver, with an anemic condition of the same part. It is probable that the dark and fugitive redness of the cellular tissue of the organ, depended upon the same cause which produced a similar injection of the kidnies, and all the abdominal veins; namely, the long agony, and the congestion of the heart.

It is stated that during life the liver was felt several inches below the right false ribs, thus giving an idea of its great size, while, after death it was found not to exceed the ordinary dimensions. The liver was specifically heavier than natural, and the effusion so elevated the false ribs, that it lost its natural support in a great measure, and fell forwards and downwards. The attempt which has been made to express in numbers the degree of insufficiency of the mitral valve, may perhaps be considered superfluous, on account of the elasticity or softness of the parts concerned. A very unpractised eye might have judged of the proportion between the mitral valve and the orifice it should have closed; but the regularity of the form of the valve was such as to warrant a belief that the proportion alluded to might be expressed in very nearly accurate terms. The result of the calculation has at least shown that the eye, in making its estimate, did not err upon the wrong side.

